# Fabrication, In Vitro and In Vivo Evaluation of Non-Ordered Mesoporous Silica-Based Ternary Solid Dispersions for Enhanced Solubility of Flurbiprofen

**DOI:** 10.3390/ph15070856

**Published:** 2022-07-12

**Authors:** Muhammad Usman Munir, Mahnoor Ikraam, Muhammad Nadeem, Syed Haroon Khalid, Sajid Asghar, Ikrima Khalid, Muhammad Irfan, Nayyer Islam, Nyla Ajaz, Ikram Ullah Khan

**Affiliations:** 1 Department of Pharmaceutical Chemistry, College of Pharmacy, Jouf University Sakaka, Aljouf 72388, Saudi Arabia; mumunir@ju.edu.sa; 2 Department of Pharmaceutics, Faculty of Pharmaceutical Sciences, Government College University Faisalabad, Faisalabad 38000, Pakistan; ikrammahnoor@gmail.com (M.I.); haroonkhalid80@gmail.com (S.H.K.); ikrima_khalid@yahoo.com (I.K.); manipharma1@gmail.com (M.I.); nayyerislam1@gmail.com (N.I.); nylaajaz@gmail.com (N.A.); 3 Department of Medicine, Xi’an Jiaotong University, Xi’an 710000, China; nadeemdogar7777777@yahoo.com

**Keywords:** flurbiprofen, gelucire, non-ordered mesoporous silica, ternary solid dispersion, gastric lesion index

## Abstract

The aim of this study was to improve the solubility and prevent the ulcerogenic effect of flurbiprofen. Initially, binary and ternary solid dispersions (BSDs and TSDs) of flurbiprofen were prepared by using non-ordered mesoporous silica and gelucire. After preformulation testing (solubility, flow properties, % yield, and entrapment efficiency), four formulations were selected for further detailed studies. Solid-state characterization of optimized formulations (S1, S6, S7, and S12) showed successful drug incorporation in the solid dispersion at the molecular state without any noticeable interactions. The in vitro solubility and release study showed an increase in solubility and 98–100% of drug release in 30–45 min. The in vivo gastro-protective effect of the optimized formulations containing flurbiprofen and silica (1:1) with 25% *w*/*w* gelucire (S6 and S12) showed a reduction in the gastric lesion index (GLI) after four days of treatment. Moreover, histological images of the stomach lining (S6 and S12) illustrated normal epithelial cells and a partially protected mucosal membrane. Thus, TSD exhibited a significant increase in solubility and the dissolution rate and reduced the gastric ulceration. Therefore, TSDs are dubbed as efficacious carriers to enhance the bioavailability of flurbiprofen while simultaneously reducing its side effects.

## 1. Introduction

Oral drug delivery systems are the most appealing carriers among all other drug delivery systems. It has many benefits, such as ease of ingestion, pain free administration, the ability to accommodate various types of active ingredients, enhanced patient compliance, ease of manufacturing, and less expensive cost as sterile conditions are not required. Despite the above-mentioned advantages, oral drug delivery systems face several issues, such as stability of the actives in highly acidic gastric environments, low bioavailability due to low solubility of drugs, poor absorption due to limited penetration of actives through mucous barriers, and drug loss due to first pass metabolism [1,2].

Use of synthetic methods and high-performance engineering methods in drug discovery have led to the production of a large number of water-insoluble drugs [3]. Currently, around 40% of the newly developed drugs are insoluble in water with the solubility <0.1 mg/mL [4,5]. Low water solubility of the drugs is a main concern as it controls the dissolution rate of drugs. Narrow dissolution rates lower the bioavailability of the drugs as drugs having solubility lower than 0.1 mg/mL exhibit dissolution-dependent absorption. In such circumstances, the dose of the drug must be increased so that the plasma drug concentration extends to the therapeutic range. In case of oral administration, this increment in drug dose results in toxicity of the gastrointestinal tract (GIT) and, hence, reduction in patient compliance. High drug concentrations increase the cost of manufacturing and also affect the product design as powders with high amounts of drug show poor flow properties [6]. Therefore, there is a need to develop solubility enhancement techniques to lower the dose and adverse effects while simultaneously increasing the oral bioavailability.

There are many techniques used for enhancing solubility of less water soluble drugs, i.e., micronization, nanosuspensions, complexation, solid dispersion, hydrotropy, co-solvency, micellar solubilization, etc. [7,8]. Solid dispersion is the low cost and widely employed technique for improving the solubility of poorly aqueous soluble drugs [9,10]. The main advantages of solid dispersion include increased solubility and dissolution profile, reduction in particle size, improved porosity and wettability, and changing crystalline drugs to amorphous states. Despite the above advantages, there are less marketed products produced by solid dispersion techniques. The lower number of marketed products is due to various issues, such as extending the production to industrial scale, instability issues in manufacturing processes, phase separation, and crystallization during storage [11]. Solid dispersions are of two types, binary solid dispersion (BSD) with a drug and one polymer and ternary solid dispersion (TSD) with a drug and two polymers. In past studies, TSD has shown better improvements in solubility, drug release, bioavailability, and stability profile of poorly aqueous soluble drugs [12].

To increase the dissolution rate of hydrophobic drugs, both hydrophilic and hydrophobic carriers were used to formulate solid dispersion. Some of the main carriers are polyethylene glycol (PEG), polyvinylpyrrolidone (PVP), urea, gelucire, eudragit, polyvinyl alcohol (PVA), crospovidone (PVP-CL), polyols, hydroxypropyl cellulose (HPC), emulsifiers, hydroxypropyl methylcellulose phthalate (HPMCP), hydroxypropyl methylcellulose (HPMC), sugars, polyacrylates, and organic acids and their derivatives [13]. Gelucires are GRAS (generally regarded as safe), lipid-based polymers that have gained fame due to their solubility enhancement and self-emulsifying properties. Gelucires belongs to the family of glycerides with PEG esters of fatty acids. There are many types of gelucires based on hydrophilic–lipophilic balance (HLB) values and melting point ranges from 33–65 °C. Gelucire 44/14 is in this class; it is an inert material with semisolid, waxy consistency that has a melting point of 44 °C and an HLB value of 14 [14]. Apart from the above-mentioned carriers, silica and its derivatives are also gaining importance in pharmaceutical research to enhance solubility and release properties of less water-soluble drugs. Mesoporous silica can be used as a carrier to formulate solid dispersion. Mesoporous silica exists in both ordered (MCM-41 and SBA-15) and non-ordered (Syloid 244FP and Syloid AL1FP) [15]. Silica is also used as adsorbent to enhance the flow properties of lipid-based solid dispersion [16].

Flurbiprofen belongs to the phenyl alkanoic acid derivatives class; it is a nonsteroidal anti-inflammatory drug (NSAID). It is used in osteoarthritis, ankylosing spondylitis, rheumatoid arthritis, acute gouty arthritis, and due to its potent analgesic effect, it is used in short-term alleviation of postoperative pain in dental patients, gingival inflammation, alveolar bone resorption in periodontal disease and as an anti-inflammatory agent in ocular disorders [17]. As a biopharmaceutical classification system (BCS) class II drug, flurbiprofen have low solubility (5–10 µg/mL) and high permeability. Due to its low solubility, it shows poor bioavailability after oral administration [18].

Literature reveals that solid dispersions of flurbiprofen were prepared by various researchers to enhance solubility and release properties, such as TSD with sodium carboxymethylcellulose (Na-CMC) and tween 80 [18], BSD dispersion with PVP [19], semi-solid dispersion with gelucire and labrasol [20], and BSD with nicotinamide [21]. However, no research study has evaluated TSD of flurbiprofen with gelucire and silica to enhance the solubility and dissolution while simultaneously reducing the gastric side effects.

The aims of this study were to enhance solubility and release properties of flurbiprofen by developing TSD with gelucire and silica by the solvent evaporation technique, comparing solubility enhancement by two types of silica, and performing extensive in vitro and in vivo analysis to check the gastro-protective effect of optimum formulations.

## 2. Results and Discussion

### 2.1. Pre-Formulation Studies

Table 1 shows the composition of the formulations, and the results of the pre-formulation studies showed that there is no significant enhancement of solubility of flurbiprofen by using 1:1, 1:3, and 1:5 of drug and silica ratios, and rather, it slightly decreased in formulation where a 1:9 drug and silica ratio was employed. Therefore, for further studies, BSD containing a 1:1 ratio of flurbiprofen and silica was chosen for the development of TSD as this ratio provided 91 times the increase in solubility of flurbiprofen when compared with the pure drug (Table 2). Different concentrations of gelucire 44/14 were used to achieve a free-flowing ternary solid dispersion. The maximum percentage for gelucire 44/14 to be included should be up to 25% of silica and drug concentration used in respective formulations. Afterwards, an agglomerated and sticky mass was obtained.

### 2.2. Physicochemical Properties

#### 2.2.1. Percentage Yield

All the binary and ternary solid dispersion preparations showed 97–99% percentage yields (Table 3). The results indicate that solid dispersions developed by the solvent evaporation method under the provided conditions gave maximum yield for each formulation. Therefore, the solvent evaporation method appears to be reproducible for the solid dispersion formulations, as previously reported [22].

#### 2.2.2. Flow Properties of Powders

Our results showed that order of flowability of solid dispersion was TSD > BSD. Furthermore, in the case of TSD, the flow properties improved with increasing concentrations of gelucire 44/14 (Table 3). These results were in good agreement with the study of Liu et al. where flowability of lipid-based drug carriers were better compared to pure mesoporous silica [23]. This might be because gelucire is a multicomponent excipient and the lipid portion provides lubrication for ease of flow of solid dispersion. In another study, the authors developed solid dispersion of flurbiprofen using gelucire 44/14 and 50/13 and found that gelucire 44/14 containing solid dispersions showed better flow properties compared to the one containing 50/13 [24]. Moreover, the results of Carr’s index and Hausner’s ratio for developed formulations were further supported by the results of angle of repose, which showed angles of 35° and 32° for formulations S6 and S12, respectively. These values indicates a good flow [25]. In a previous study, phosphatidylcholine-based dispersions of aprepitan were developed, and the angle of repose lied in the range of 31.54 to 34.7, indicating good flow properties [26].

#### 2.2.3. Estimation of Drug Content

The drug content was used to determine the amount of flurbiprofen entrapped in the binary and ternary solid dispersions. The results showed that with increasing concentrations of gelucire 44/14, entrapment efficiency was enhanced. This is probably because silica carriers can better trap hydrophobic molecules in porous structures in the presence of gelucire 44/14. Gelucire not only traps flurbiprofen but also supports interactions with silanol groups present on the surface of Syloid. Secondly, the flurbiprofen to carrier components ratio is increased, which prevents drug leakage (Table 3). ML336 is a recently developed chemical inhibitor of Venezuelan equine encephalitis virus. To overcome its limited solubility and stability, lipid-coated mesoporous silica nanoparticles were developed. By coating, the drug was retained in the carrier in a high quantity, and furthermore, it enabled an enhanced circulation time and biocompatibility [27]. In BSD, it was observed that formulation S7 entrapped more of the drug as Syloid AL1 has more surface area (605 m^2^/g) compared to Syloid 244 (379 m^2^/g), which is present in formulation S1 [28]. In the literature we found that [29] investigated loading capacities of celecoxib, cinnarizine, and paracetamol in five different grades of Sylysia^®^ with varying surface areas, pore diameters, and pore volumes. They reported higher drug loading with increasing surface area and decreasing pore volume of mesoporous silica carrier.

#### 2.2.4. Solubility Studies

The solubility of all binary and ternary solid dispersions were determined at pH 6.8 (PBS) and 1.2 (0.1 N HCl) and distilled water to assess solubility in intestinal, gastric, and neutral pH.

**Figure 1:**
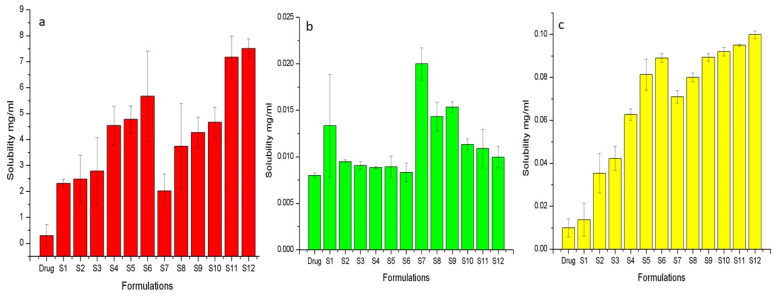
Solubility of drug at (**a**) pH 6.8 (PBS), (**b**) pH 1.2 (0.1 N HCl), and (**c**) distilled water.

At pH 6.8 (Figure 1a), Syloid AL1 FP showed more increase of solubility in TSD (S12) containing 25% gelucire 44/14, that is 218.34 times compared to Syloid 244 FP (S6). TSD of Syloid 244 FP containing 25% gelucire 44/14 (S6) showed 180 times an increase in solubility when compared with the pure drug. This was due to the higher surface area of Syloid AL1 FP, i.e., 605 m^2^/g compare to 379 m^2^/g for Syloid 244FP [28]. The higher surface area enhances wettability and, hence, solubility of the drug [30]. Furthermore, as drug molecules are confined within the porous network, drugs with low solubility and crystalline nature are converted to their amorphous counterparts, thus exhibiting higher solubility. At pH 6.8, as the concentration of gelucire 44/14 is increased, solubility is also enhanced. Gelucire is a non-ionic water dispersible surfactant that enhances drug solubility by enhancing wettability of the drug, the formation of micellar structures, and the reduction in drug particle size.

At an acidic pH (Figure 1b), binary solid dispersions showed slightly higher solubility compared to the pure drug. However, with the addition of gelucire 44/14 in the ternary solid dispersion, solubility started decreasing. These results gave us a clue that the addition of gelucire 44/14 in the ternary solid dispersion can provide a gastro-protective effect, as flurbiprofen is prone to cause gastric ulcers. The coating of silica particles with gelucire 44/14 retards the release of the drug from the carrier at pH 1.2, as gelucire 44/14 is a multicomponent excipient, where glyceride fractions are digested slowly [31].

Syloid AL1 FP showed higher solubility of flurbiprofen in distilled water (Figure 1c). BSD of Syloid AL1 FP (S7) showed eight times an increase in solubility compared to BSD of Syloid 244 FP (S1) that showed only 1.3 times an increase in solubility. TSD of Syloid AL1 FP with 25% gelucire (S12) showed 10.6 times an increase in solubility, while TSD of Syloid 244 FP with 25% gelucire (S6) showed 8.9 times an increase in solubility. The Syloid AL1 containing formulation showed more increase in solubility because of its higher surface area (605 m^2^/g for Syloid AL1 vs. 379 m^2^/g for Syloid 244). This higher surface area and the presence of gelucire enhances wettability and, hence, increased solubility of the drug [30].

Based on the solubility analysis, the optimum formulations selected were S1, S6, S7, and S12. Formulations S1 and S7 were selected to compare the effect of BSD, and formulations S6 and S12 were selected to compare the effect of TSD on solubility and the release of flurbiprofen.

### 2.3. Solid State Characterisation

#### 2.3.1. FTIR

**Figure 2:**
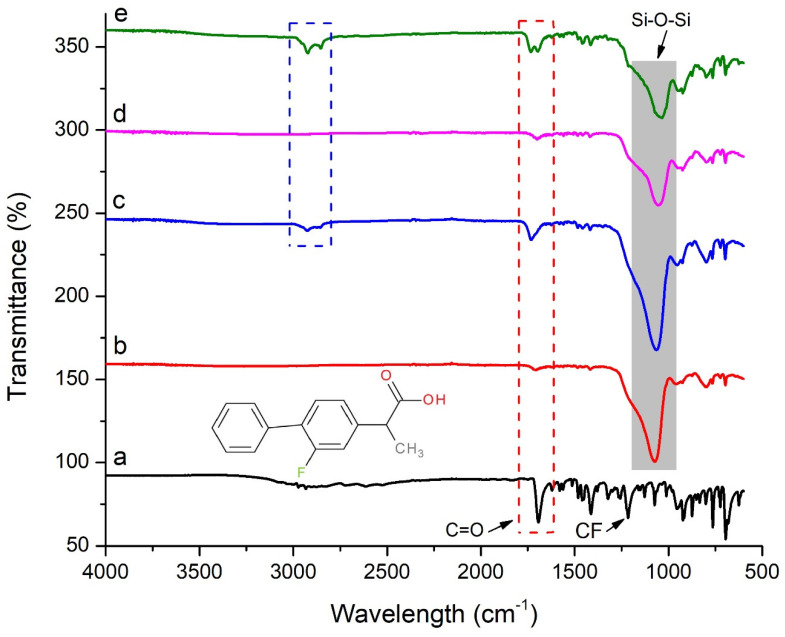
FTIR spectrum of (**a**) flurbiprofen, (**b**) S1, (**c**) S6, (**d**) S7, and (**e**) S12.

FTIR is widely employed to detect functional groups and assess the interaction between drugs and pharmaceutical carriers. The FTIR spectrum of pure flurbiprofen (Figure 2a) showed a peak at 1701 cm^−1^, which is due to carboxyl group (C=O) stretching and a peak at 1220 cm^−1^ due to (C-F) stretching [32]. All the formulations showed characteristic peaks at particular positions that show successful entrapment of flurbiprofen in the carriers. Additionally, the intensity of the carbonyl group stretching peak at 1701 cm^−1^ was reduced in solid dispersion formulations in comparison to the pure drug, which indicates an interaction between the drug and the carrier. The literature widely reports on such interactions between the drug and mesoporous silica via hydrogen bonding when it is adsorbed within the pores [33]. However, this interaction is weaker and reversible due to the fact that flurbiprofen was recovered completely when dissolution/release studies were performed [20]. The peaks at 1065 cm^−1^ in solid dispersion formulations were due to the siloxane (Si-O-Si) stretching vibration [34]. Formulations S6 and S12 (Figure 2c,e) also show characteristic peaks of gelucire 44/14 at 2920 and 2856 cm^−1^ for the C-H stretch [35]. Thus, it is inferred that no major interaction exists between the drug and the formulation excipients.

#### 2.3.2. XRD

**Figure 3:**
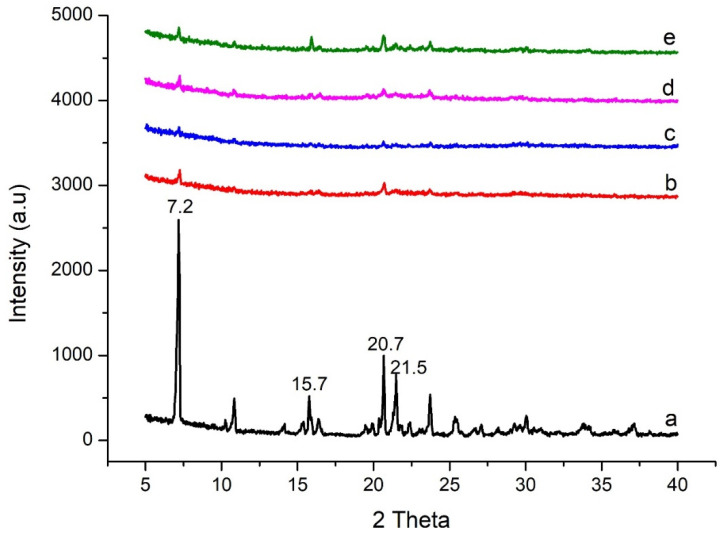
(**a**) Flurbiprofen, (**b**) S1, (**c**) S6, (**d**) S7, and (**e**) S12.

XRD is a rapid and nondestructive technique to acquire distinctive patterns for crystalline materials. The XRD graph of flurbiprofen (Figure 3a) showed numerous distinctive diffraction peaks with high intensities, which depict the crystalline nature of the drug. However, all the XRD graphs of BSD and TSD were showing diffused peaks that indicate flurbiprofen was successfully entrapped in the pores of mesoporous silica in the amorphous state [36]. In the literature, it is mentioned that the mesoporous silica material prevents the recrystallization of entrapped active ingredients. Furthermore, it is suggested that all entrapped drugs would remain in the amorphous form if the pore size of the carrier is twelve times smaller than the molecular size of the drug [33]. The XRD results hinted at the development of amorphous dispersions, which are widely employed for enhancement of solubility and dissolution of drug.

#### 2.3.3. SEM

**Figure 4:**
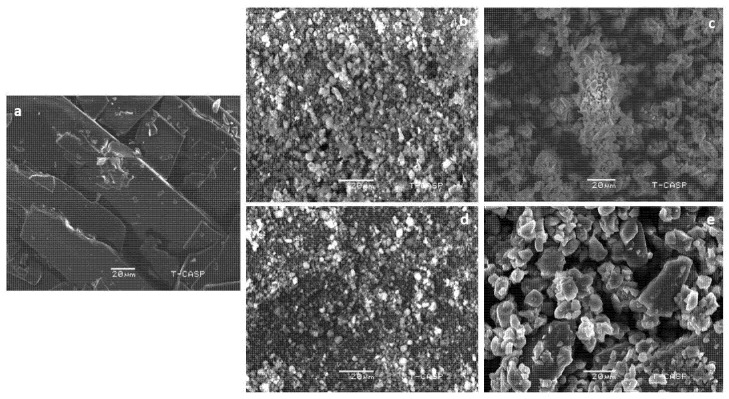
SEM images of (**a**) flurbiprofen, (**b**) S1, (**c**) S6, (**d**) S7 and (**e**) S12.

To observe the differences in the surface morphologies of the pure drug and the solid dispersions, their SEM images were taken. In the SEM images of pure flurbiprofen, rectangular crystals were observed [37], showing their crystalline nature (Figure 4a). However, in BSD with Syloid 244 (Figure 4b), the surface of silica particles was spherical and smooth, showing most of the drug was captured within the silica pores. In the case of BSD with Syloid AL1 (Figure 4d), the surface was not that smooth, indicating some of the drug might have adsorbed on the surface of the silica pores. This can be due to the fact that Syloid AL1 have smaller pore diameters and large surface areas compared to Syloid 244 [28,38]. The surface of TSD prepared by Syloid 244 was also smooth (Figure 4c), showing that Syloid 244 entrapped all the drug and gelucire 44/14 due to large pore diameters. Nevertheless, some of the gelucire 44/14 and drug particles were visible in SEM images of TSD of Syloid AL1 (Figure 4e), making them less smooth. In all the cases, most of the drug was entrapped in the silica particles in amorphous form [34] and are also in agreement with XRD results described in previous section. The presence of the drug in amorphous form are helpful to enhance solubility and dissolution.

### 2.4. In Vitro Release

**Figure 5:**
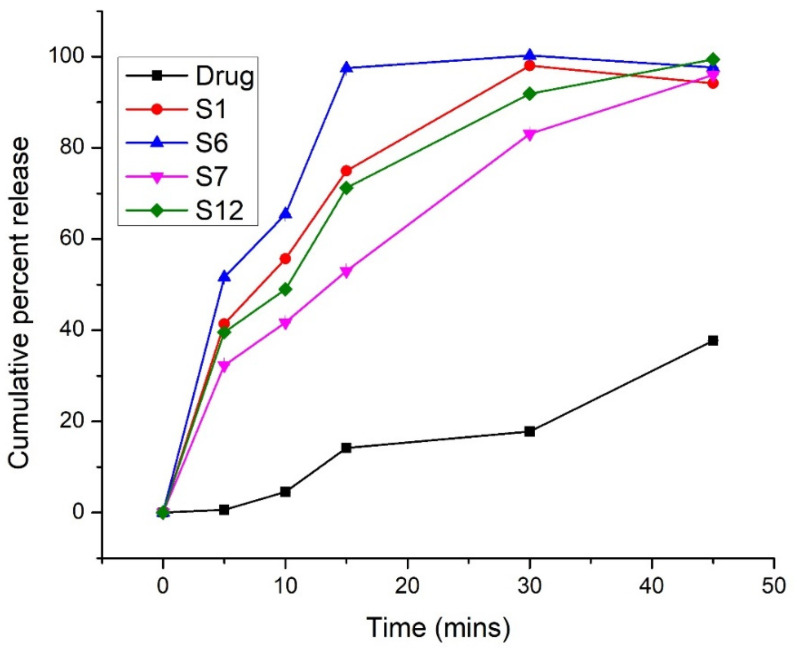
Dissolution profile at pH 6.8 (PBS).

**Figure 6:**
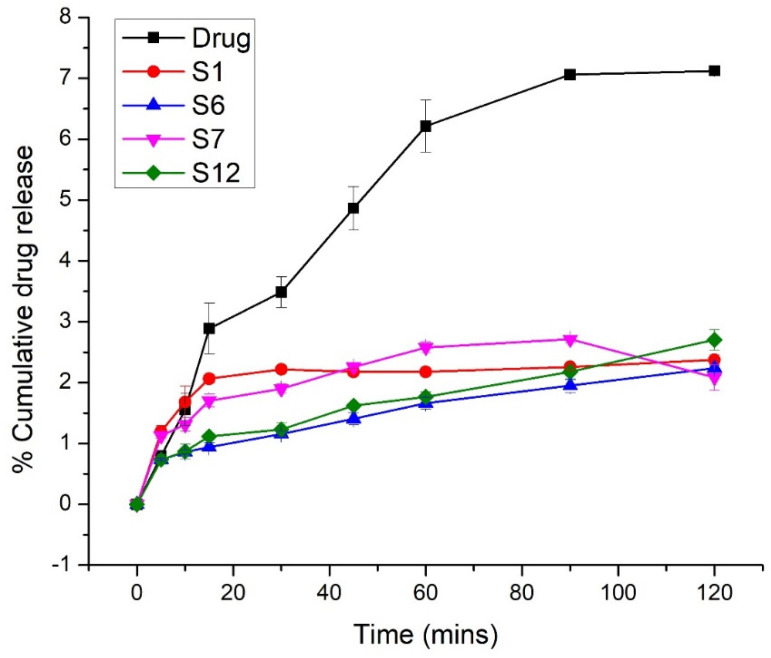
Dissolution profile at pH 1.2 (0.1 N HCl).

The results of the in vitro release study at pH 6.8 (PBS) are shown graphically in Figure 5, which shows that Syloid 244 FP with 25% gelucire 44/14 (S6) showed maximum drug release that is 100% in the first 30 min. TSD of Syloid AL1 FP with 25% gelucire 44/14 (S12) showed 99% drug release in the first 45 min. The trend of drug release was S6 > S12 > S7 > S1 > pure drug. The pure drug only showed 37% release in 45 min. Overall, Syloid 244 FP based solid dispersions showed enhanced dissolution and faster release because of the large pore volume and large pore diameters of Syloid 244 compared to Syloid AL1 [28]. On the other hand, the drug incorporated within the pores of silica in amorphous form also paved the way for dissolution enhancement of flurbiprofen. As shown by the results of FTIR, the weak interaction between flurbiprofen and silica also increased the drug dissolution to some extent [39]. The results of TSD were better compared to BSD, which showed that the combination of two carriers could enhance dissolution and solubility of BCS class II drugs [40]. At the acidic pH, the dissolution profiles of TSDs of both Syloid 244FP and Syloid AL1FP showed decreased drug release compared to the pure drug and BSDs (Figure 6). In 120 min, the pure drug, S1, S6, S7, and S12 showed 7.12%, 2.37%, 2.24%, 3.19%, and 2.70% drug release, respectively. This decrease in drug release at the acidic pH overcomes the gastric side effects of pure flurbiprofen. Pure flurbiprofen showed pH dependent solubility and release. At the acidic pH, the solubility and release of flurbiprofen are reduced [20]. Moreover, the presence of gelucire 44/14 could also provide a gastro-protective coating to reduce its side effects [24,41].

### 2.5. Estimation of Gastro-Protective Effect In Vivo

#### 2.5.1. Macroscopic Scoring

**Figure 7:**
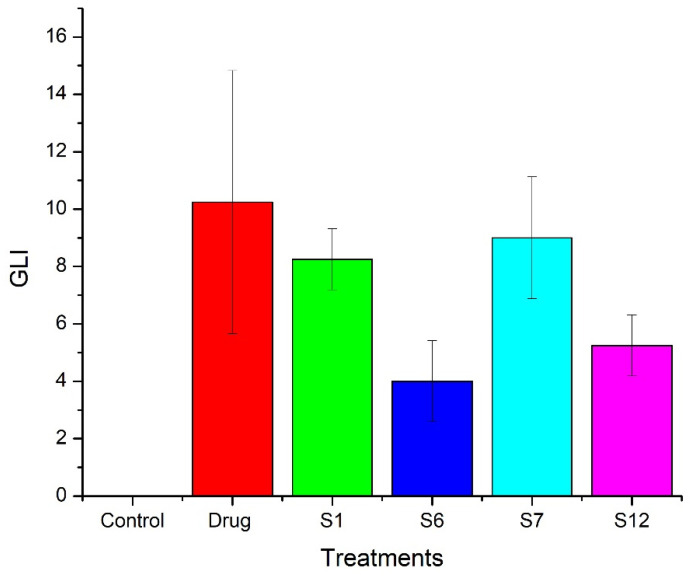
GLI in gastric mucosa of rats after different treatments.

Shown in Figure 7 is the trend of the average gastric lesion index of the pure drug (10.25 ± 4.59) > S7 (9 ± 2.12) > S1 (8.25 ± 1.06) > S12 (5.25 ± 1.06) > S6 (4 ± 1.41) > control (0).

**Figure 8:**
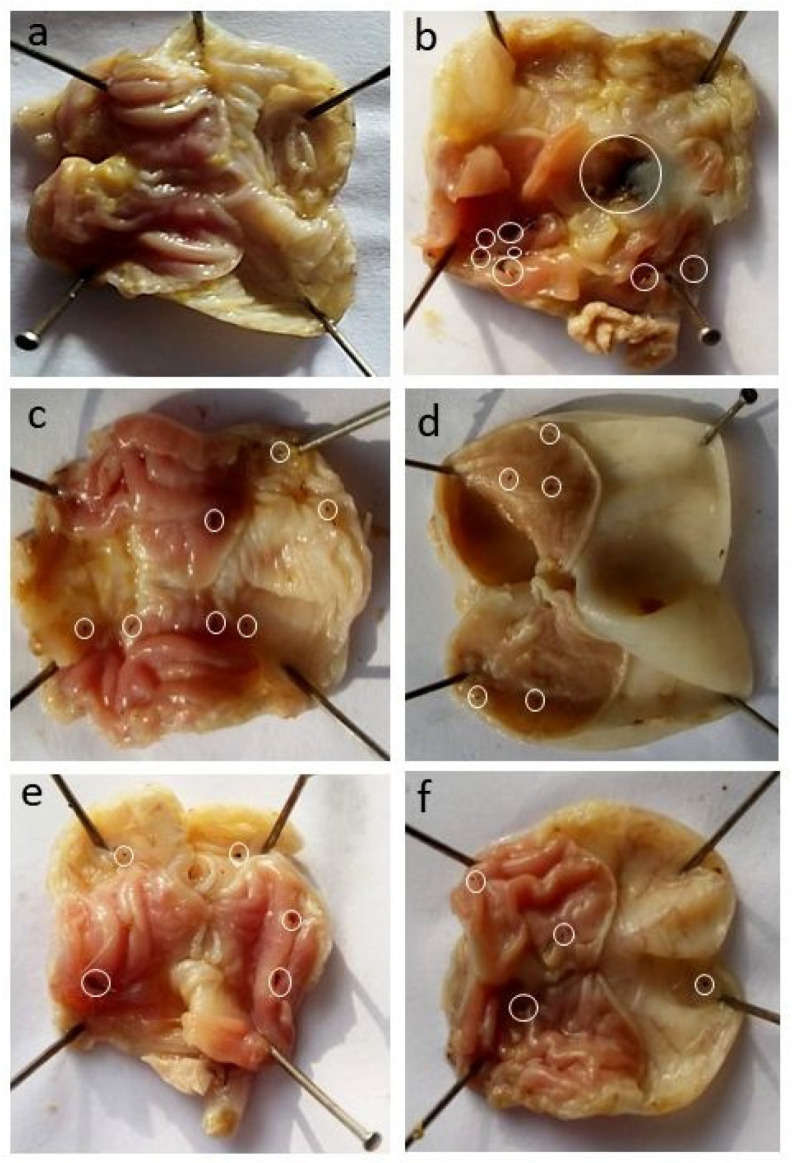
Macroscopic lesion in stomach mucosa of rats: (**a**) control, (**b**) flurbiprofen, (**c**) S1, (**d**) S6, (**e**) S7, and (**f**) S12.

According to GLI, the TSD formulations showed less lesion scores compared to BSD and the pure drug. BSD did not protect the gastric mucosa because the silica can cause mucosal damage. The silanol groups in silica develop hydrogen bonding with cellular membranes and, thus, causes hemolysis [42]. Gelucire 44/14 in TSD is majorly responsible for protecting the gastric mucosa from the side effects of the drug and silica. Macroscopic images of individual stomachs from each group are shown in Figure 8. Gastric lesions are most prominent in the pure drug treated group (Figure 8b). Flurbiprofen belongs to the NSAIDs group and is known for inducing ulcers and bleeding in GIT. Thus, our strategy to develop TSD of flurbiprofen will simultaneously address the solubility and side effects of flurbiprofen.

#### 2.5.2. Microscopic Study

**Figure 9:**
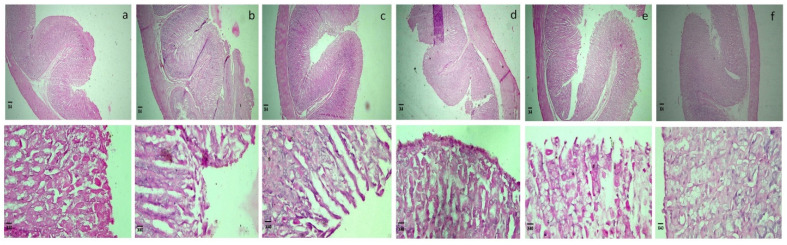
Histological micrographs of (**a**) control group, (**b**) group treated with pure flurbiprofen, (**c**) group treated with S1, (**d**) group treated with S6, (**e**) group treated with S7, (**f**) group treated with S12 at 4× and 40×.

The histopathological micrographs of rats treated with pure flurbiprofen and various BSDs and TSDs are shown in Figure 9. The specimen from the control group (Figure 9a) showed an intact mucosal layer with a normal histological pattern of the rat’s stomach mucosa and normal epithelial cells [43].

The figures of the specimen of the rat’s stomach from the group treated with pure flurbiprofen (Figure 9b) showed a damaged epithelium and necrotic lesions penetrated in mucosa. Oedema of submucosa is also seen [43]. Flurbiprofen inhibits cyclooxygenase enzyme (COX-1 and COX-2). Prostaglandins (PGs) produced by COX-1 are responsible for maintaining gastric mucosa and provide gastric protection, while PGs produced by COX-2 are involved in enhancing vessel dilatation and permeability and pain and fever associated with inflammation. Therefore, administered flurbiprofen results in gastric lesions, bleeding, and epithelial damage by inhibiting COX-1 enzymes [44]. The stomach histology of rats treated with S1 (Figure 9c) showed a disruption of the mucous epithelium and necrotic lesions in mucosa [45,46]. Here, Syloid 244 FP alone is not protecting the gastric mucosa from gastric side effects of flurbiprofen. Syloid 244 may cause membranolysis by reaction of silanol group of silica with membranes that damage the cell membranes (necrosis) or cause cell death (apoptosis) [42]. The specimens of the rat’s stomach treated with S6 formulation (Figure 9d) showed partially intact mucosa and normal epithelial cells [46]. Here, gelucire 44/14 provided a gastro-protective effect to our formulation by preventing direct contact of the drug and silica with the gastric mucosa [41,47]. There was a complete loss of mucosal layer and a lot of fibrin deposition in the mucosal specimen of the rat treated with S7 formulation (Figure 9e) [45,46]. Syloid AL1 FP is also damages the mucosal membrane through the interaction of the silanol group with the membrane. The histopathological analysis of the specimen of the rat’s mucosa treated with the S12 formulation (Figure 9f) showed an intact mucosa, and this protection was provided by gelucire 44/14 [41,47].

## 3. Materials and Methods

### 3.1. Materials

Flurbiprofen (CAS number: 510449-4, molecular weight 244.26 gm/mol) was received as a gift from Axis Pharmaceuticals, Faisalabad, Pakistan; Lauroyl polyoxyl-32 glycerides (gelucire 44/14, CAS number: 121548-04-7) was kindly donated by Gattefossè, Lyon, France. Non-ordered mesoporous silica (Syloid 244FP EU and Syloid AL1/63 FP EU; CAS number: 7631-86-9) was received as a gift sample from Grace Discovery Sciences, Chicago, IL, USA. Monobasic potassium phosphate (CAS number: 7778-77-0) was purchased from Daejung Reagents Chemicals and Metals Co., Ltd., Siheung, Korea. Sodium hydroxide (NaOH), ethanol, dichloromethane, and hydrochloric acid were of analytical grade and used as received.

### 3.2. Preparation of Solid Dispersion

Solid dispersions were prepared by the solvent evaporation technique with few minor modifications, as reported previously [40]. Dichloromethane (DCM) was used as a solvent. Briefly, an accurately weighed amount of flurbiprofen (Table 1) was initially dissolved in DCM in a glass mortar on a magnetic stirrer and was followed by the addition of gelucire 44/14 with constant stirring. When gelucire was dissolved, a specified amount of Syloid was added to this solution. This suspension was magnetically stirred for 30 min at 300 rpm. After that, the mortar was placed in a hot air oven at 38 °C for overnight drying. After drying, the solid dispersion was triturated with a glass pestle and sieved through sieve number 40. The prepared solid dispersion was stored in airtight glass vials and placed over a bed of silica beads in a desiccator until further analysis.

### 3.3. Pre-Formulation Studies

In the pre-formulation studies, the binary solid dispersions of flurbiprofen, Syloid 244FP and Syloid AL1FP, are prepared in ratios of 1:1, 1:3, 1:5, and 1:9. The solubility of each formulation was checked in pH 6.8 (PBS) in triplicates, and their means are taken to check times increase in solubility by formulation to that of pure flurbiprofen. Ternary solid dispersions of flurbiprofen, silica, and gelucire 44/14 are prepared in 1:1:1, 1:1:0.5, and 1:1:0.25 ratios, and their physical appearances were checked to gain a free-flowing solid dispersion.

### 3.4. Physicochemical Properties

#### 3.4.1. Practical Yield

Percentage yield refers to the ratio of actual yield to theoretical yield. Final weight of every formulation was divided by sum of actual weights of all the ingredients added [38].

$$\mathrm{Practical}\;\mathrm{yield}\;\%\;=\frac{\mathrm{Actual}\;\mathrm{yield}}{\mathrm{Theoretical}\;\mathrm{yield}}\times 100$$

#### 3.4.2. Properties of the Powder

##### Density

Bulk and tapped densities of formulations are measured, as reported in literature. An amount of 1 gm of formulation was taken in a graduated cylinder to measure volume [23]. It was calculated by the following formula:

$$\mathrm{Bulk}\;\mathrm{density}=\;\frac{\mathrm{Mass}}{\mathrm{Volume}}\;$$

The density powder was tapped 100 times to allow the powder volume to plateau [23]. It was calculated by the following formula:

$$\mathrm{Tapped}\;\mathrm{density}=\frac{\mathrm{Mass}}{\mathrm{Tapped}\;\mathrm{Volume}}\;$$

##### Flowability

Flow properties of powder were determined by Carr’s index, the Hausner ratio, and the angle of repose by the following equations [23]:

$$\mathrm{Carr}’s\;\mathrm{index}\;=\;\frac{\left(\mathrm{Tapped}\;\mathrm{density}-\mathrm{Bulk}\;\mathrm{density}\right)}{\mathrm{Tapped}\;\mathrm{density}}\;\mathrm{Hausner}\;\mathrm{ratio}\;=\;\frac{\mathrm{Tapped}\;\mathrm{density}}{\mathrm{Bulk}\;\mathrm{density}}\;$$

##### Angle of Repose

To determine the angle of repose, 5 gm of each formulation were taken and allowed to flow through a funnel with an orifice diameter of 10 mm. The funnel was placed 4 cm above the surface of a paper. When powder passed through the funnel, a cone-like pile was formed. The radius (r) and height (h) of the pile was measured, and the angle of repose was calculated as follows [48]:

$$\mathrm{Angle}\;\mathrm{of}\;\mathrm{repose}\;(\theta )=\tan^{-1}\;\;\frac{\mathrm{height}\;\left(h\right)\;}{\mathrm{radius}\;\left(r\right)}\;$$

#### 3.4.3. Estimation of Drug Content

Solid dispersion equivalent to 10 mg of drug was taken in 10 mL of ethanol in a conical tube and vortexed. A total of 1 mL of above solution was further diluted with ethanol and assayed spectrophotometrically at 247 nm by using the previously developed calibration curve. Results are expressed in drug content (drug entrapped) [49].

$$\%\;\mathrm{Drug}\;\mathrm{content}=\frac{\mathrm{Actual}\;\mathrm{amout}\;\mathrm{of}\;\mathrm{drug}\;\mathrm{calculated}}{\mathrm{Amount}\;\mathrm{of}\;\mathrm{drug}\;\mathrm{intially}\;\mathrm{added}}\times 100\;$$

#### 3.4.4. Solubility Studies

Excess of flurbiprofen and solid dispersion was added in 3 mL of solvent (6.8 pH phosphate saline buffer (PBS), 1.2 pH (0.1 N HCl), and distilled water). They were placed in a water bath shaker at 37 °C for 72 h, centrifuged at 6000 rpm for 30 min, and clear supernatant 1 mL was taken, diluted, and assayed spectrophotometrically at 247 nm to determine the drug dissolved per ml [37]. Solubility was determined in 6.8 pH (PBS), 1.2 pH (0.1 N HCl), and distilled water.

### 3.5. Solid State Characterization

#### 3.5.1. Fourier-Transform Infrared Spectrometry (FTIR)

ATR-FTIR spectroscopy was used for confirming the structure of the drug and its interaction with carrier components. FTIR spectra of the pure drug and solid dispersions (S1, S6, S7, S12) were obtained by scanning in the range of 4000–500 cm^−1^ [20].

#### 3.5.2. X-ray Diffraction (XRD)

Crystallinity of pure flurbiprofen and solid dispersion preparations (S1, S6, S7, S12) were studied by the powder X-ray diffraction technique [50].

#### 3.5.3. Shape and Surface Morphology

The shape and morphology of pure flurbiprofen and flurbiprofen-loaded solid dispersions (S1, S6, S7, S12) were examined using scanning electron microscopy (SEM) [37].

### 3.6. In Vitro Release

Release of flurbiprofen from optimum formulations were observed in 0.1 N HCl, pH 1.2, and pH 6.8 (PBS) at 37 °C and 100 rpm paddle speed using a USP dissolution apparatus (Pharmatest, Hainburg, Germany). The pure drug and solid dispersion equivalent to 20 mg of drug were taken in a bucket with 500 mL release media. A total of 5 mL of sample was withdrawn from the release media at 5, 10, 15, 30, 45, 60, 90, and 120 min, and equivalent fresh release medium was replaced. The content of flurbiprofen was assayed directly at 247 nm by using a UV-visible spectrophotometer [51]. Release studies were performed identically in triplicates, and means was taken.

$$\%\mathrm{age}\;\mathrm{drug}\;\mathrm{released}=\frac{\mathrm{Concentration}\;\mathrm{of}\;\mathrm{drug}\;\mathrm{released}}{\mathrm{actual}\;\mathrm{drug}\;\mathrm{concentration}}\times 100$$

After the release studies, the Krosmeyer–Peppas model was applied to release the data to elucidate the mechanism involved in the drug release [52].

### 3.7. Estimation of Gastro-Protective Effect

The animal study was approved by the Institutional Ethical Review Committee (ERC) of the Government College University Faisalabad, Faisalabad Pakistan (Ref. No: GCUF/ERC/2019-625). In this study, albino rats weighing 150–200 g were selected to compare the ulcerogenic potential of pure flurbiprofen and the optimum solid dispersion formulations. For this test, the purchased rats were acclimatized for oneweek in the in-house animal facility at a relative humidity (55 ± 5%) and temperature (25 ± 2 °C) and 12 h of the light/dark cycle using the standard rodent diet and water ad libitum. Healthy rats were divided into six groups each containing six rats (n = 6) and were housed in a separate cage to prevent them from being injured by other rats. The animals were fasted overnight with a free supply of water. The solid dispersions of samples S1, S6, S7, and S12 and the pure drug were administered orally at a dose of 5 mg/kg suspension in 0.5% *w*/*v* carboxymethyl cellulose. The controlled group was given only 0.5% *w*/*v* carboxymethyl cellulose suspension. The dose was given for 4 consecutive days, and the animals were sacrificed on the fourth day after four hours of dosing [53].

#### 3.7.1. Macroscopic Scoring

The stomachs of the sacrificed animals were separated and cleaned with isotonic saline solution and were cut along the greatest curvature. The ulcers were measured using digital Vernier calipers. The arbitrary scores (ASs) given to the ulcers were 0 for no ulcer/lesion, 0.5 for one or more ulcers of length <1 mm, 1 for ulcers/lesions of length 1–2 mm, and 2 for ulcers/lesions with length >2 mm. The arbitrary scores were multiplied with the number of lesions to determine the lesion index as follows [54]:

Gastric lesion index (GLI) = AS × No. of ulcers/lesions

#### 3.7.2. Microscopic Analysis

All the stomachs of the animals were preserved in 20% *v*/*v* formalin buffer solution for histological analysis. Tissue sections of animals were observed under a microscope fitted with a 5 megapixel camera (Accuscope 3000, Commack, NY, USA) to determine the extent of ulceration and mucosal damage [54].

### 3.8. Statistical Analysis

Statistical analysis of the results was carried out by employing either Student *t* test or one-way ANOVA with post-hoc Tukey test. Significance was established for the differences where *p*-value was less than 0.05.

## 4. Conclusions

To conclude this study, TSDs of flurbiprofen were prepared using gelucire 44/14 and two types of silica, i.e., Syloid 244FP and Syloid AL1FP. The concentration of gelucire 44/14 was tested from 5 to 25% to obtain a free-flowing TSD. Solid-state characterization hinted dispersion of flurbiprofen at the molecular level, and no harmful interaction of the formulation ingredients were observed. TSD of both silica grades successfully increased the solubility and dissolution of flurbiprofen at pH 6.8. The addition of gelucire 44/14 in the TSD formulations prevented the release of flurbiprofen in the acidic media (1.2 pH). The in vivo gastro-protective study also revealed that gelucire 44/14 also reduced the ulcerogenic effect of flurbiprofen and silica. We can conclude that the combination of Syloid 244FP or Syloid AL1FP and gelucire 44/14 (S6 and S12) were successful in increasing the solubility and dissolution of flurbiprofen.

## Figures and Tables

**Table 1:** Composition of binary and ternary solid dispersions.

Sr. No.	Code	Grade of Silica	Drug: Silica (*w*/*w*)	Gelucire 44/14 (*w*/*w*)
1	S1	Syloid 244 FP	1:1	---
2	S2	Syloid 244 FP	1:1	5%
3	S3	Syloid 244 FP	1:1	10%
4	S4	Syloid 244 FP	1:1	15%
5	S5	Syloid 244 FP	1:1	20%
6	S6	Syloid 244 FP	1:1	25%
7	S7	Syloid AL1 FP	1:1	---
8	S8	Syloid AL1 FP	1:1	5%
9	S9	Syloid AL1 FP	1:1	10%
10	S10	Syloid AL1 FP	1:1	15%
11	S11	Syloid AL1 FP	1:1	20%
12	S12	Syloid AL1 FP	1:1	25%

**Table 2:** Preformulation studies for selection of flurbiprofen silica ratio.

Sr. No.	Code	Composition	Solubility (mg/mL) in pH 6.8 (PBS)	Times Increase in Solubility
1	Flurbiprofen	----	0.03	----
2	SA	Flurbiprofen:Syloid 244FP (1:1)	2.74	91
3	SB	Flurbiprofen:Syloid 244FP (1:3)	3.10	103
4	SC	Flurbiprofen:Syloid 244FP (1:5)	3.34	111
5	SD	Flurbiprofen:Syloid 244FP (1:9)	2.94	98
5	SE	Flurbiprofen:Syloid Al1FP (1:1)	2.75	92
6	SF	Flurbiprofen:Syloid Al1FP (1:3)	3.16	105
7	SG	Flurbiprofen:Syloid Al1FP (1:5)	3.23	108
8	SH	Flurbiprofen:Syloid Al1FP (1:9)	3.01	100

**Table 3:** % yield, entrapment efficiency, and flow properties of BSD and TSD.

Sr. No.	Code	% Yield	% Drug	Bulk Density gm/mL	Tapped Denstiy gm/mL	Carr’s Index	Hausner’s Ratio	Angle of Repose (°)	Remarks
1	S1	99	80.72	0.178	0.278	35.71	1.56	61	Very poor
2	S2	98.4	93.24	0.178	0.263	32.14	1.47	56	Very poor
3	S3	98	97.10	0.192	0.263	26.92	1.36	52	Poor
4	S4	98.8	98.74	0.208	0.263	20.84	1.26	45	Passable
5	S5	98.2	99.30	0.208	0.25	16.67	1.2	39	Fair
6	S6	97.6	99.79	0.217	0.25	13.04	1.15	35	Good
7	S7	99.5	87.31	0.5	0.769	35	1.53	59	Very poor
8	S8	98.8	89.74	0.454	0.714	36.36	1.57	57	Very poor
9	S9	97.5	91.00	0.5	0.714	30	1.42	51	Poor
10	S10	98	93.68	0.5	0.667	25	1.34	43	Passable
11	S11	98.7	99.47	0.34	0.4167	20	1.25	37	Fair
12	S12	99	80.72	0.38	0.4167	14.28	1.67	32	Good

## Data Availability

Data is contained within the article.
